# Women’s experiences of medical treatment for endometriosis and its impact on PRE-EMPT trial participation: a qualitative study

**DOI:** 10.1186/s40814-018-0358-5

**Published:** 2018-11-05

**Authors:** Elaine Denny, Annalise Weckesser, Georgina Jones, Stavroula Bibila, Jane Daniels, Siladitya Bhattacharya, on behalf of the PRE-EMPT team

**Affiliations:** 1 Centre for Social Care, Health and Related Research, Faculty of Health, Education and Life Sciences, Birmingham City University, Westbourne Rd, Edgbaston, Birmingham B15 3TN UK; 2 School of Social Sciences, Leeds Beckett University, Leeds, LS1 9HE UK; 3 Coventry University Group, Armstrong Siddeley Building, Gosford St., Coventry, CV1 5DL UK; 4 Nottingham Clinical Trials Unit, Queen’s Medical Centre, C Floor, South Block, Nottingham, NG7 2UH UK; 5 Institute of Applied Health Sciences, School of Medicine, Polwarth Building, Foresterhill, Aberdeen, AB25 2ZD UK

**Keywords:** Endometriosis, Treatment, Experiences, Qualitative research, Trial recruitment

## Abstract

**Background:**

Endometriosis is a common cause of chronic pelvic pain which can relapse after surgery, yet little research has been conducted on women’s experience of medical treatments for prevention of recurrence and the influence of this on participation in clinical trials.

**Methods:**

This study explored women’s past experiences with medical treatments for endometriosis symptoms and the impact this has on their motivation to enter the pilot phase of a post-conservative surgery clinical trial, PRE-EMPT: Preventing Recurrence of Endometriosis by Means of long acting Progestogen Therapy. Qualitative methodology was adopted, involving semi-structured interviews in three UK cities, and one focus group was used to collect data from women with a diagnosis of endometriosis participating in the PRE-EMPT trial.

**Results:**

Ten women were interviewed individually and four took part in the focus group discussion. Women’s willingness to enter the PRE-EMPT trial was bound up with their previous experiences, present situation and future expectations of medication, as well as the control offered by flexible randomisation which allows the option to reject a particular treatment post-surgery.

**Conclusion:**

Women were strongly influenced by previous experience and personal circumstances in their decision to enter the PRE-EMPT trial. This decision was facilitated by the ability to ‘opt out’ of the treatment arm(s) they found unacceptable. This element of choice offered patients a sense of control in the randomisation process and has important implications for clinical trial design and recruitment.

**Trial registration:**

ISRCTN97865475. EUDRACT number 2013–001984-21.

## Background

Endometriosis is a chronic condition in women where endometrial tissue found outside the uterus produces an inflammatory response [1]. It has often been called the enigmatic disease; its aetiology is not clearly understood [2, 3], symptoms are extremely variable and there is only weak evidence underpinning many common medical treatments [4–6]. While some women with laparoscopic evidence of pelvic endometriosis experience extreme pain, fatigue and infertility, others remain asymptomatic [5] and the severity of symptoms is not correlated with the extent of disease [7]. Many qualitative studies have been undertaken that seek to gain insight into the experience of endometriosis, but a search using the relevant databases for qualitative studies on health and health care revealed no study where the primary purpose was to explore women’s experiences of medical treatments for endometriosis. However, this is an important issue for many symptomatic participants in endometriosis research. Women describe in graphic detail the extent of their pain and the impact it has on their lives, often living with the condition for many years before receiving a diagnosis [8–12]. They report going through cycles of ineffective medical treatments [9, 12–14], often with unpleasant side effects [7, 10, 15, 16]. Many of these treatments are also contra-indicated in women who wish to become pregnant. The lack of effective treatments will sometimes lead women to abandon medical treatment altogether [8] or to seek self-management strategies through alternative and complementary therapy [17].

Laparoscopic excision of endometriotic lesions is considered to be effective in pain outcomes, in comparison to diagnostic surgery alone [18], but the evidence is weak and risks of relapse and reoccurrence of symptoms are high [19]. Prevention of recurrence of pain symptoms following surgery involves the use of agents which reduce circulating levels of oestrogen, causing shrinkage of remaining endometriotic deposits and prevention of de novo lesions. A number of drugs, including long-acting reversible contraceptives (LARCs) are in current use, but there is no consensus as to which is most effective and cost effective (see latest trial protocol: https://njl-admin.nihr.ac.uk/document/download/2011878). There is a need, therefore, to assess the clinical effectiveness of the available post-surgical treatment options in terms of controlling the recurrence of endometriosis and improving quality of life. The PRE-EMPT trial (see Table 1) aims to evaluate the clinical effectiveness, cost effectiveness and acceptability of such treatment in women following conservative surgery for endometriosis pain. The pilot phase of this trial [20] allowed flexible randomisation (i.e. the ability of participants to opt out of one or two treatments, as long as one LARC and one non-LARC was accepted) between four post-surgical options in preventing the recurrence of endometriosis:

1. Combined oral contraceptive pill (COCP), containing 30 μg ethinylestradiol and 150 μg levonorgestrel taken either continuously or cyclically;
2. Levonorgestrel intrauterine system (LNG-IUS), as a 20-μg per day formulation;
3. Depot medroxyprogesterone acetate (DMPA), as a 150-mg injection every 12 weeks;
4. No medical treatment initiated post-laparoscopy.

**Table 1:** The PRE-EMPT trial protocol summary

Design	A randomised, pragmatic multicentre trial with integrated economic evaluation
Setting	Up to 40 NHS hospitals within the UK
Target population	Women of reproductive age, who are undergoing laparoscopy to investigate whether their pelvic pain is due to endometriosis
Exclusion criteria	Current infertility, immediate plans to conceive
Health technologies assessed	The main comparison is long-acting reversible contraception (LARC) versus combined oral contraceptive pill (COCP). Participants can have a pre-randomisation choice of LARC (or alternatively one will be randomly allocated): i) Levonorgestrel-releasing intra-uterine system (LNG-IUS) (fitted by a gynaecologist) or ii) 3 monthly depot medroxyprogesterone acetate (DMPA) injections (administered by the patient’s gynaecologist or general practitioner); subcomparisons will be stratified by this choice
Outcomes	The primary outcome is the recurrence of symptoms as evaluated by the pain domain of the Endometriosis Health Profile–30 (EHP-30) questionnaire at 36 months post-randomisation. The EHP-30 is a validated, responsive health-related quality of life measure for endometriosis. It will also be assessed prior to randomisation and at 6, 12 and 24 months. Secondary outcomes: • All other symptom and quality of life (QoL) domains of the EHP-30 • Non-menstrual pelvic pain and dysmenorrhea measured by 0–100 visual analogue (VAS) pain scale • Fatigue, as measured by the Fatigue Severity Score • Menstrual regularity • Generic QoL (EQ-5D) and capabilities, as measure of wellbeing (ICE-CAP) • Further diagnostic and therapeutic surgery for endometriosis (as a proxy for recurrence) • Discontinuation rates of randomised treatment, with reasons for change, serious adverse events • Cost per quality-adjusted life year (QALY) and cost per change in symptom score. • An increased knowledge of issues identified as important by the participants regarding their treatment and its impact on their lives
Analysis	The main comparison will be LARC vs COCP, with sub-comparisons of the groups where the intention is to treat with either LNG-IUS or DMPA if randomised to LARC. The primary outcome will be analysed using a linear regression model including a variable for each treatment group and including baseline score and the minimisation factors as covariates. Effect sizes will be presented as point estimates and 95% confidence intervals. Standard statistical methods will be used for other outcomes. All analysis will be by intention to treat.
Sample	The study will have 90% power (*p* = 0.05) to detect an 8-point difference in the main comparison assuming the standard deviation of the EHP-30 pain domain is 22 points. This will require 160 women per group, 320 in total. To account for 20% loss to follow-up, this target has been inflated to 400 women in total

As part of the pilot study for the PRE-EMPT trial, a stand-alone qualitative study was undertaken. Participation in clinical trials, in particular randomised controlled trials (RCTs), has always been problematic, with many failing to recruit anticipated numbers [21]. Both quantitative and qualitative studies have considered why this is the case, but their findings have had little effect on overall recruitment [22]. This article will present the findings from the qualitative study within the PRE-EMPT clinical trial which had the aim of exploring women’s previous experience of medical treatments for endometriosis symptoms and the impact this has on their motivation to enter a clinical trial post conservative surgery.

## Methods

### Design

A qualitative approach to the study was considered appropriate as the aim of the study was to gain insight into women’s experiences and motivations and also to explore issues of importance to them rather than to adhere strictly to a script. Narratives are an important way for people to explain disruptive events in their lives, and the use of narrative interviews and a focus group in this study allowed women to reflect on living with endometriosis and to raise the issues that had greatest impact on their lives [23]. Three sites taking part in the PRE-EMPT trial, Aberdeen Royal Infirmary, Birmingham Women’s Hospital and Edinburgh Royal Infirmary, all in the UK, took part in the qualitative study, which comprised a focus group discussion and individual interviews (see Table 2). The focus group and semi-structured interview topic guides were informed by available literature on women’s experiences of medical treatments for endometriosis symptoms, as well as the expertise of the PRE-EMPT Trial Management Group. As a means of establishing rapport, at the beginning of interviews and the focus group, some demographic data were collected.

**Table 2:** Focus group and interview schedules

1. Past medical treatment experiences • Tell me about the types of medical treatments you have tried • Prompts: Why did you try [names of treatments]? Who influenced your decision? What were your expectations? • Tell me about your experiences of …. [names of treatments] • Prompts: Effectiveness/ineffectiveness of treatment? How long effective for? Side effects? 2. Views on medical treatments offered in PRE-EMPT After undergoing surgery, there are four possible treatments—how do you feel about: • ‘The pill’ • ‘The coil’ • ‘Depo Provera’ • No treatment • [For each treatment above] Prompt: (Un)Acceptable? Why? Past experiences? Future hopes? Do you think the treatment would be more effective post-surgery? • Were there any treatments that you would *not* accept? Which? Why? 3. Views on medical trials [General] • What do you think about medical trials? • What do you think about randomisation? • Prompts: Understandings of randomisation/how treatment is allocated. Randomisation acceptable to you? Is the possibility of not getting treatment acceptable? 4. Views on participation in PRE-EMPT trial • What do you think about the PRE-EMPT trial? • Prompts: Hopes for the trial? Concerns about the trial? • Why did you take part in the PRE-EMPT trial? • Prompts: What did you hope to gain from participating? What were your concerns about participating? • What would be a barrier to you participating? • Prompts: Personal factors? Time/travel costs? Trial factor? Concerns about treatment availability/randomisation? • Did you have a preference for which arm you would be randomised to? Why? Why not? • Is this a worthy trial? Why? Why not? • How do you feel about the length of the trial (3 years)? 5. Concluding questions • Is there anything we did not discuss that you would like to talk about? • Do you have any questions for me?	

Favourable ethical approval for this study was obtained from the North of Scotland Research Ethics Committee and site-specific permission from the NHS Trusts of each of the hospitals involved.

### Recruitment

Purposive sampling, that is sampling people who are relevant to the research question [24], was undertaken. All of the women recruited had been randomised in the PRE-EMPT trial and had given additional consent for the stand-alone qualitative study following discussion with the local research nurses. They were then approached by the researchers (AW and SB) and asked to take part in either the focus group (if from Aberdeen) or an individual interview. Formal written consent was obtained by the research nurses prior to interviews and the focus group; researchers reiterated consent verbally on the day of interview/focus group. The focus group and interviews were carried out by AW and SB, both experienced qualitative researchers with an interest in women’s reproductive health. To foster open and honest discussions, the researchers explicitly stated that they were university academics separate from hospitals involved in the trial and reinforced the confidential and anonymous nature of women’s participation. In appreciation of their time, participants were given a £20 gift voucher at the end of their interview or the focus group.

### Data collection

The focus group discussion took place at Aberdeen Royal infirmary. Initially, six women agreed to take part, but only four attended. The focus group discussion elicited women’s past experiences with the treatments included in the trial and examined their willingness to accept each treatment post-surgery and whether their inclusion in any of them would constitute a barrier to continuation within the study. Three women were interviewed in person (from Birmingham) and seven were interviewed over the telephone (from Edinburgh and Aberdeen) (*n* = 10). Telephone interviews offered a flexible means to include women from a wide geographical spread. Individual interviews allowed respondents to feel more relaxed and able to address issues concerning their endometriosis treatment experiences that may have been too sensitive to discuss in a focus group setting [25]. The focus group and interviews were digitally audio recorded with consent and fully transcribed verbatim. Written consent was obtained to use anonymised extracts from the transcripts in publications. The focus group lasted 1 h and the interviews were 25 to 60 min in duration. All data were collected in January and February 2015.

### Analysis

Thematic analysis [24] was carried out by the qualitative research team, which consisted of two qualitative leads (ED and GJ) and two research assistants (AW and SB). AW and SB independently read the transcripts line by line, identifying emergent themes and created initial codes. AW and SB brought these codes together to create a coding framework, and AW coded the transcripts with NVIVO 10. AW employed constant comparison, an iterative method of analysis, searching for each themed code throughout the entire data set (focus group, interview and telephone interview data), comparing all instances until no new themes were identified. Saturation was deemed to have been reached when no new themes were emerging from additional interview data. ED and GJ each independently read proportions of the transcriptions. The team jointly discussed and agreed upon common patterns and broader themes from women’s experiences and perspectives on treatment acceptability. Dissident views and areas of diversity among the team were also considered and resolved. To establish the trustworthiness [26] of the analysis, the four researchers independently read the transcripts and compared findings from the two parts of the data collection process. Although generalisation is not an aim of qualitative research, we were able to show consistency with the existing literature on women’s experience of symptomatic endometriosis.

## Results

In reporting this study, standards for reporting qualitative research (SRQR) were adopted [27] (see Table 3). Women participating in both the focus group and individual interviews shared their views and experiences of medical treatments and their motivation for enrolling in the PRE-EMPT trial. As no novel treatment was on offer, many women had previous experience of treatments available as part of the RCT (either as endometriosis treatment or for contraceptive purposes), which strongly influenced their acceptability.

**Table 3:** Standards for reporting qualitative research (SRQR) https://www.ncbi.nlm.nih.gov/pubmed/24979285

	Page/line no(s).
Title and abstract	
Title - Concise description of the nature and topic of the study Identifying the study as qualitative or indicating the approach (e.g., ethnography, grounded theory) or data collection methods (e.g., interview, focus group) is recommended	P1
Abstract - Summary of key elements of the study using the abstract format of the intended publication; typically includes background, purpose, methods, results, and conclusions	P1
Introduction	
Problem formulation - Description and significance of the problem/phenomenon studied; review of relevant theory and empirical work; problem statement	P1/30-P3/3
Purpose or research question - Purpose of the study and specific objectives or questions	P2/31-P3/3
Methods	
Qualitative approach and research paradigm - Qualitative approach (e.g., ethnography, grounded theory, case study, phenomenology, narrative research) and guiding theory if appropriate; identifying the research paradigm (e.g., postpositivist, constructivist/ interpretivist) is also recommended; rationale**	P3/6-P3/30 P8/12-16
Researcher characteristics and reflexivity - Researchers’ characteristics that may influence the research, including personal attributes, qualifications/experience, relationship with participants, assumptions, and/or presuppositions; potential or actual interaction between researchers’ characteristics and the research questions, approach, methods, results, and/or transferability	P3/46-50
Context - Setting/site and salient contextual factors; rationale**	P3/55-70
Sampling strategy - How and why research participants, documents, or events were selected; criteria for deciding when no further sampling was necessary (e.g., sampling saturation); rationale**	P3/34-54
Ethical issues pertaining to human subjects - Documentation of approval by an appropriate ethics review board and participant consent, or explanation for lack thereof; other confidentiality and data security issues	P3/26-29 P10/34-38
Data collection methods - Types of data collected; details of data collection procedures including (as appropriate) start and stop dates of data collection and analysis, iterative process, triangulation of sources/methods, and modification of procedures in response to evolving study findings; rationale**	P3/52-P4/4
Data collection instruments and technologies - Description of instruments (e.g., interview guides, questionnaires) and devices (e.g., audio recorders) used for data collection; if/how the instrument(s) changed over the course of the study	P3/69-P4/4 P 3 Table 2
Units of study - Number and relevant characteristics of participants, documents, or events included in the study; level of participation (could be reported in results)	P4/44-49 P3 Table 4
Data processing - Methods for processing data prior to and during analysis, including transcription, data entry, data management and security, verification of data integrity, data coding, and anonymization/de-identification of excerpts	P3/69-P4/4
Data analysis - Process by which inferences, themes, etc., were identified and developed, including the researchers involved in data analysis; usually references a specific paradigm or approach; rationale**	P4/6-32
Techniques to enhance trustworthiness - Techniques to enhance trustworthiness and credibility of data analysis (e.g., member checking, audit trail, triangulation); rationale**	P4/24-31
Results/findings	
Synthesis and interpretation - Main findings (e.g., interpretations, inferences, and themes); might include development of a theory or model, or integration with prior research or theory	P4/50-58 P4/80-P6/4 P6/12-P7/48 P7/57-P8/10
Links to empirical data - Evidence (e.g., quotes, field notes, text excerpts, photographs) to substantiate analytic findings	P4/60-P7/88
Discussion	
Integration with prior work, implications, transferability, and contribution(s) to the field - Short summary of main findings; explanation of how findings and conclusions connect to, support, elaborate on, or challenge conclusions of earlier scholarship; discussion of scope of application/generalizability; identification of unique contribution(s) to scholarship in a discipline or field	P8/11-P9/73 P9/100-P10/15
Limitations - Trustworthiness and limitations of findings	P9/117-30
Other	
Conflicts of interest - Potential sources of influence or perceived influence on study conduct and conclusions; how these were managed	P10/58-60
Funding - Sources of funding and other support; role of funders in data collection, interpretation, and reporting	P10/20-23

**The rationale should briefly discuss the justification for choosing that theory, approach, method or technique rather than other options available, the assumptions and limitations implicit in those choices, and how those choices influence study conclusions and transferability. As appropriate the rationale for several items might be discussed together

### Sample

The ages of the 14 participating women varied from 19 to 36 years, and experience of symptoms of endometriosis varied from 2.5 to 16 years. There was a range of symptomology, treatment histories and allocated treatment groups within the trial. A table detailing overall baseline characteristics of the sample is provided in Table 4.

**Table 4:** Baseline characteristics of sample

		(*n* = 14)
Age, years	Mean (SD)	27.9 (5.7)
Ethnic group, *n* (%)	White British	12 (86)
	Black/Black British Caribbean	1 (7)
	Asian/Asian British Pakistani	1 (7)
	Missing	–
Parity, *n* (%)	0	12 (86)
	1	1 (7)
	2	1 (7)
	Missing	–
Employment status, *n* (%)	Full-time	10 (72)
	Part-time	1 (7)
	Unemployed	1 (7)
	Student	2 (14)
	Missing	–
Previous treatment experiences with LNG-IUS, DMPA or COCP, *n* (%)	All	2 (14)
	LNG-IUS and COCP	1 (7)
	DMPA and COCP	5 (36)
	COCP	6 (43)
	Missing	–
Stage of endometriosis, *n* (%)	I	5 (35)
	II	4 (29)
	III	4 (29)
	IV	1 (7)
	Missing	–
Number of previous laparoscopies, *n* (%)	0	7 (50)
	1	5 (36)
	2	2 (14)
	Missing	–
Extent of excision as judged by surgeon, *n* (%)	Complete	11 (79)
	Missing	–
EHP-30 pain score at baseline	Mean (SD)	59.7 (9.7)
	Missing	–

### Personal circumstances and past experiences

Most women in both the FG and interviews spoke of past experiences of one or more of the interventions offered by the trial. Although some women had only recently received a diagnosis, nearly all had a long history of symptoms and had received at least one treatment.

Women described how endometriosis was affecting all aspects of their lives, particularly work and personal relationships.

And then it was just getting unbearable, like difficult to walk. And then sex with my now ex-boyfriend was awful, which probably contributed to the end of the relationship. (P7, interview).

But I think it was just more I was getting to the point that I had to cancel nights out, days out, holidays and everything because the pain was just [unbearable] (P1, Focus Group).

Women graphically described the pain they experienced and the strategies that they would adopt to manage everyday life.

Because you have the pain all the time, but then there’s that other pain, and you brace yourself - is the only way I can describe it. But I would literally start running through my work diary thinking, ‘Can I manage this day? Is there anything I can move around? Can I make it to the end of the week?’ I would have real panic thinking, ‘I’m not going to make it through today, I’m going to have to cancel clinics.’ I think that’s the bit that took over my life. (P4, Focus Group).

A number of women spoke of being dismissed and not taken seriously in the past and of being misdiagnosed, which added to delays in receiving treatment. For them, the trial was a sign that ‘someone was taking an interest’ in endometriosis, which encouraged them to participate.

Women had complex views in regard to whether treatments they had used prior to surgery would be more effective post-surgery. For example, some women (*n* = 3) had negative past experiences with both the COCP and DMPA. While they would not accept the latter post-surgery because of unpleasant side effects, they were willing to accept the COCP as they believed it may be more effective post-surgery. ‘I know [the pill] didn’t help in the past, but, you know it might change now. Since I’ve had an operation, it might help’ (P5, interview). Other women (*n* = 3) did not believe a particular hormonal treatment would have more efficacy post-surgery as they had undergone surgeries before and found the treatment(s) equally ineffective or negative after. ‘I didn’t really think I would have a different experience [with hormonal treatments] because I’ve had the surgery before’ (P7, interview).

Current life circumstances, particularly in relation to reproduction, also influenced decision making regarding acceptability of treatment options.

I do not want a child right now, so it’s quite good that I am on the pill and that’s monitoring the endometriosis and that side of things as well. … So I think that’ll vary for everyone. I mean, I think, you know, depending on what age you are and what age you want to have children or where you are going in life, career-wise and things as well, I think that will vary. (P8, interview).

### Views on individual treatment options

The majority of participants reported previous experience with one or more of the medications on offer in the trial. No one option, which included the combined COCP, LNG-IUS, DMPA, and no treatment, was found to be more or less acceptable by participants. This was similar to results of the clinical trial pilot study, which found that no particular treatment combinations were chosen more than others [20].

When women found the COCP an unacceptable treatment option, this was due to experience of negative side effects in the past (including weight gain, mood swings and erratic periods) or because it had been ineffective in the management of their symptoms. As one woman states, ‘[The pill] didn’t help at all. I was getting my periods, like, quite a lot more than I should have. And it didn’t help with the pain’ (P4, interview). Those who found the pill acceptable did so because of success with it as a treatment option in the past, the ease with which they could both start and discontinue the pill, and a need for contraception.

Some women expressed ambivalence about the acceptability of COCP. For example, one participant found that it was not completely effective at controlling her endometriosis symptoms in the past, but she was willing to accept it as a treatment option post-surgery as she regarded it as the best of the choices available. Furthermore, on the COCP, she states that ‘the pain still comes and goes, but the bleeding is a lot more under control. I feel like now I control [my endometriosis] when I start and stop the pill as opposed to [endometriosis] controlling my life’. (P8, interview). Other women who had limited success with the COCP in the past were willing to accept it as a treatment option because they would be prescribed a different contraceptive pill to the one(s) previously taken or they believed that post surgery, the pill may be more effective.

Women did not accept LNG-IUS as a treatment option if they had previous negative side effect experiences (including discomfort, cramping, weight gain, increased bleeding, poor fittings and infections) or because of negative experiences of friends and family members, who had usually used it for contraceptive purposes. For example, one woman rejected LNG-IUS as her mother had become pregnant while using it. Those who found LNG-IUS acceptable did so because they had no previous experience with it and/or appreciated its convenience as a long-acting treatment, as once the coil was inserted, ‘you could forget about it’ (P5, interview).

Participants who would not accept DMPA as a treatment option had found it ineffective in the management of their symptoms and/or had highly negative experiences (including heavier/more frequent periods and migraines) in the past. Two women found the repeat visits required to receive the injections inconvenient, as one states ‘I work, I go to college, I’m trying to have as much of a life as I can when I’m not in pain...The thought of going to the doctor, it’s quite far away from me as well… I just don’t think it would be for me’ (P5, interview). Those who accepted the DMPA as a treatment option did so because it was a new option they had no previous experience with and were therefore open to trying it.

Women who found randomisation to non-treatment unacceptable did so as they were concerned their endometriosis symptoms would return more quickly post-surgery or because they required hormonal contraception. One woman enrolled in this arm of the trial expressed concern that although happy with this option now, her life circumstances may change and she could require a hormonal treatment for contraceptive purposes on becoming sexually active. Those who found non-treatment acceptable reported feeling they were willing to let their body ‘have a break’ (P6, interview) from hormonal treatments and to ‘let [their] body settle’ (P5, interview) after surgery and to assess the efficacy of the surgery in relation to their symptoms.

### Decision making regarding treatments and views on randomisation

Women found randomisation acceptable as they had an element of choice over which treatment groups to be randomised to (see Table 1).

I obviously have tried pretty much every treatment for endometriosis before surgery. So that would obviously have a big bearing on me. If something’s not worked before, you know, you would as much as [pauses], like say, if a doctor told me to take something just to try it, of course I would want to. But I think sometimes you know your body better … especially if you’ve had [the treatments] in the past. (P8, interview)

Many women viewed randomisation as part of a ‘trial and error’ process that is necessary to learn which treatment options work best for individual women as ‘every [treatment] works differently with everyone’s body’ (P4, interview). Half of the participants (*n* = 7) reported that without the option to opt out of a particular treatment group (or groups), they would have declined trial participation. Two women viewed randomisation positively as it relieved them from the burden of choosing a treatment without adequate knowledge of the options. Randomisation was seen as ‘quite a good thing’, by one such participant as it ended the stress of deciding between multiple options and the confusion of ‘other people telling you what to have, putting you off things, it’s better just the doctor saying, “That’s what you’re having.” Try it and that’s it’ (P5, interview).

### Expectations of trial participation

Women chose to participate in the trial for reasons of altruism and self-interest. Participants expressed a desire to help others with endometriosis and to prevent them ‘suffering’ from the same physical, emotional and health consequences they had experienced. Many hoped the trial would ‘raise awareness’ and shorten pathways to diagnosis, even though these were not aims of the trial. ‘It took me a long time to be diagnosed… I wouldn’t like other people to go through the wait and the pain that I went through’ (P6, interview). Women viewed the trial, and receiving medical treatment post-surgery, as a means of ‘gaining control’ over their condition so that they could ‘get their life back’. Women also reported enrolling in the trial out of ‘desperation’ and a willingness to ‘try anything’ to manage the condition.

Overwhelmingly, women found the 3-year length of their participation acceptable as this long study reflects the chronic nature of endometriosis and the unpredictable nature of symptoms that vary between women and over different stages of an individual’s life. Further, they viewed the relatively long time period as a positive both for themselves as individuals, as the efficacy of their post-surgery treatment would be monitored over the course of 3 years, as well as for the overall success of the trial. Women felt it took time for their bodies to ‘get used to’ hormonal treatments (or to the absence of hormonal treatment) and for negative side effects to subside. Some of those who had undergone previous laparoscopic surgery for endometriosis felt that this alone reduced pain for 2 to 3 years. Thus, the trial length was seen as advantageous to allow both for the efficacy of post-surgery treatments to be considered in light of this ‘adjustment period’ and for the decline in the effectiveness of surgery to reduce pain over time. Only one woman expressed a concern about the trial length, stating that some participants may wish to become pregnant during this time and would thus need to withdraw from the study. Others indicated that they did not view their participation as irrevocable, as a change in personal circumstances could precipitate a change in treatment decisions.

## Discussion

The aim of the study was to explore women’s experience of medical treatments for endometriosis symptoms and the impact this has on their motivation to enter a clinical trial post conservative surgery. This trial aimed to compare existing treatments; no novel therapy was being offered and so previous and present experience of these was particularly relevant. Our findings are consistent with those of McCann et al. who conducted two metasyntheses of studies which considered reasons for participation in phase 3 trials over the time periods 1996–2005 and 2005–2010. From these, they identified four influences on the decision to enter an RCT [22]. In the first study, (1996–2005) personal circumstances around the time of recruitment to the study, views of the trial treatment interventions, views of the trial processes and procedures, and weighing up benefit to self and others all influenced the decision making of prospective participants. From the findings of the second synthesis (2005–2005), McCann et al. argued that the first of these influences—where the participant was situated in terms of ‘health status, treatment juncture and perceptions of these at the time of recruitment’—mediated ‘the nature and salience of judgements’ about the other three [22], page 238. In addition, we have demonstrated how willingness to enter the PRE-EMPT trial was bound up with previous experience and future expectations of medication, as well as the control offered by the option to reject a particular treatment post-surgery. Overall, women did not report one post-surgery treatment option (including the no-treatment group) as more unacceptable than any others. However, individually, women had strong feelings about which treatments they found objectionable based on their past negative experiences, or those of their friends and family members, with that particular treatment. These strong treatment preferences shape participants’ views on randomisation, with half indicating they would have declined the trial if they had not been allowed the element of choice over which trial options they would (and would not) be randomised to. Similarly, research on women’s participation in a trial comparing surgical techniques for stress incontinence found that non-participants were not averse to clinical trials per se but had strong preferences about specific aspects of treatment, in particular for a specific product and a general anaesthetic [28].

While women’s previous experience of treatment influenced notions of acceptability of different treatments, poor experience did not necessarily equate with unacceptability. Women’s reasoning was complex, with past experience being weighed against future expectation of treatment in their decision making. Some women felt that their changed circumstances (i.e. following surgery) could lead them to experiencing a more positive result or changes to the medication itself (e.g. a different COCP) may result in fewer side effects. That women were prepared to retry medical treatments that may have been unsuccessful or had limited success in the past is a key additional finding. This openness to receive treatments that have previously been ineffective speaks to the ‘desperation’ some women reported in their ongoing struggles to find effective interventions and desire to ‘try anything’ to relieve their condition.

Qualitative thematic analysis highlighted that the complex rationales for which treatment options women found acceptable need to be understood within the context of their ongoing struggle to find long-term, effective medical treatments that address their endometriosis symptoms. Existing quantitative research shows only weak evidence of the effectiveness of many medical treatments for managing endometriosis pain [4]. Additionally, a Cochrane review for endometriosis found only low to moderate evidence for many medical treatments, including suppression of menstrual cycles through GnRH analogues, LNG-IUS and Danazol, confirming the limited effectiveness of commonly prescribed interventions [6] Although our study only asked women about their experience of the treatment options in the trial, the analysis highlights the patients’ perspective in navigating this myriad of often ineffective medical treatments available. It demonstrates that the complexity of women’s reasoning for wholly rejecting some treatment options, while sometimes accepting others they also had previous negative experiences with, is based on their ongoing attempts with multiple medical treatments, each with varied efficacy over time.

Past qualitative research has shown that prior to diagnosis, women report enduring their endometriosis symptoms for years (7–11) and ‘suffer at a physical, emotional and social level’ when they remain undiagnosed [9]. Within this body of literature, it has also been noted that women may be taking medications that are often used to treat endometriosis, and this can mask symptoms of the condition and contribute to delayed diagnosis [9, 10, 12]. These delays occur both at an individual patient level and a medical level [9, 14] and commonly feature the normalisation of women’s pain [9, 14]. Post-diagnosis, qualitative studies of endometriosis experiences show that women report limited treatment success and recurrent symptom relapses [9, 12–14], creating what has been termed a ‘medical merry-go-round’ [29]. In relation to these findings, our analysis demonstrates that the willingness of participants in this trial to try, and retry, treatments out of ‘desperation’ likely stems from the hardships women face in delayed pathways to endometriosis diagnosis and the subsequent merry-go-round of medical treatments. To what extent women’s experiences of the medical merry-go-round foster these feelings of desperation, and shape their treatment-seeking practices, warrants further empirical investigation. Women’s experiences of ongoing cycles of treatment efficacy and inefficacy are also reflected in findings on participants’ views of the PRE-EMPT trial itself. Women made sense of the length of their trial participation (3 years) in terms of their own benefit as well as of the success of the overall trial. Women’s experience of the long-term nature of endometriosis and the short- or medium-term effect of treatments was seen as good justification for a long follow-up period.

This study sheds light on the rationales underpinning patients’ decisions to enter clinical trials, which may be helpful to future studies. The ability to recruit and retain suitable participants to research, and in particular to randomised trials, remains a major concern [21]. Qualitative methods used early in the research process have been shown to improve recruitment, by addressing concerns that are identified by participants in preparatory work, in order to increase the relevance and acceptability of participating in RCTs [30]. This study demonstrates that researchers would benefit from considerations of the complex ways in which participants’ past histories, current circumstances and future hopes with treatments offered in a trial comes to influence acceptability. Women in this trial were open to retrying treatments with which they had past negative or ambivalent experiences, especially in light of changed circumstances (in this case, the potential for the increased efficacy of treatments post laparoscopic surgery). Such findings are useful for recruitment strategies and can potentially inform how women are offered various treatment arm options. They also demonstrate how the processes by which the research is conducted may be designed to offer an element of choice and/or control to participants.

Results from this qualitative study also highlight the importance of increased choice offered to patients through the option to ‘opt out’ of a particular treatment arm (or arms). This element of choice offered patients an important sense of control in the randomisation process and is important to consider where there are strong preferences among potential trial recruits. Past qualitative research has repeatedly shown that the issue of control is particularly important to women living with endometriosis, as it has been described as a disease that causes individuals to feel a loss of control over their bodies and their lives [10, 11, 17, 29, 31]. Furthermore, this research highlights that women view taking action through medical (as well as complementary) treatments as a means of reclaiming lost control [11, 17]. Thus, our analysis demonstrates that women actively engaged in the management of their endometriosis could potentially find randomisation into a trial without the ability to choose between treatment arm(s) as unacceptable loss of control over their condition. Knowledge of the importance of this element of choice to patients can also be considered in the design the clinical trials and help improve overall recruitment. Flexible trial designs have proved beneficial in other trial situations, allowing multiple questions to be simultaneously addressed [32, 33]. However, flexibility is often provided to accommodate clinician preferences or practice, rather than those of the participants.

### Strength and limitations

As far as we are aware, this is the first qualitative study to have experience of medical treatments for endometriosis as its main focus. Using a qualitative research design has enabled us to gain insight into this experience and how women make sense of participation in a clinical trial within this context. Using two research methods has increased methodological rigour: however, a focus group of under six participants is below the generally agreed minimum [24]. The main limitation of the study is that participants were drawn only from women who had agreed to be randomised to the PRE-EMPT trial. Women who declined to participate, the two women who did not attend the FG and women who are successfully managed with medication may have different perspectives on living with endometriosis. Research into why people with long-term conditions decline to take part in trials is limited but include not wanting disruption to their lives, for example by extra clinic visits, or their state of health at the time of being approached [34], and the unknown of a clinical trial [35].

PRE-EMPT trial participants are to be followed up for 3 years, and during this time, women are likely to be on conception-preventing drugs. It is therefore probable that this would be an additional reason women for whom fertility is a key issue would not be represented in the study.

## Conclusion

Women in this study demonstrate complex pathways to decision making regarding which treatments for endometriosis they find acceptable, and this shapes their willingness to enter a clinical trial for post-surgery treatments to prevent symptom reoccurrence. In addition to a mix of altruism and self-interest, women entered the PRE-EMPT trial because of the ability to ‘opt out’ of treatment arm(s) they found unacceptable. This element of choice offered patients a sense of control in the randomisation process and has important implications for clinical trial design and recruitment. By not only including participants in the early stages of research design but also by allowing them to articulate their own decision-making processes in assessing treatment options and trial participation, researchers in many areas of health research may optimise recruitment and retention. In terms of endometriosis policy, it needs to be acknowledged that many women have been on a long journey, and their accumulated knowledge of what works, what does not and at what personal cost needs to be included at the micro and macro levels of policy making.
